# The thermal limits of cardiorespiratory performance in anadromous Arctic char (*Salvelinus alpinus*): a field-based investigation using a remote mobile laboratory

**DOI:** 10.1093/conphys/coaa036

**Published:** 2020-04-23

**Authors:** Matthew J H Gilbert, Les N Harris, Brendan K Malley, Adrian Schimnowski, Jean-Sébastien Moore, Anthony P Farrell

**Affiliations:** 1 Department of Zoology, University of British Columbia, #4200-6270 University Blvd, Vancouver, BC, V6T 1Z4, Canada; 2 Freshwater Institute, Fisheries and Oceans Canada, 501 University Crescent, Winnipeg, MB, R3T 2N6, Canada; 3 Arctic Research Foundation, 1505 Charleswood Road, Winnipeg, MB, R3S 1C2, Canada; 4 Institut de Biologie Intégrative et des Systèmes and Département de Biologie, Université Laval, 1030 Avenue de la Médecine, Quebec City, QC, Québec G1V 0A6, Canada; 5 Faculty of Land and Food Systems, University of British Columbia, #4200-6270 University Blvd, Vancouver, BC, V6T 1Z4

**Keywords:** Arctic char, Arctic fisheries, cardiorespiratory, climate change, fish physiology, thermal tolerance

## Abstract

Despite immense concern over amplified warming in the Arctic, physiological research to address related conservation issues for valuable cold-adapted fish, such as the Arctic char (*Salvelinus alpinus*), is lacking. This crucial knowledge gap is largely attributable to the practical and logistical challenges of conducting sensitive physiological investigations in remote field settings. Here, we used an innovative, mobile aquatic-research laboratory to assess the effects of temperature on aerobic metabolism and maximum heart rate (*f*_Hmax_) of upriver migrating Arctic char in the Kitikmeot region of Nunavut in the central Canadian Arctic. Absolute aerobic scope was unchanged at temperatures from 4 to 16°C, while *f*_Hmax_ increased with temperature (*Q*_10_ = 2.1), as expected. However, *f*_Hmax_ fell precipitously below 4°C and it began to plateau above ~ 16°C, reaching a maximum at ~ 19°C before declining and becoming arrhythmic at ~ 21°C. Furthermore, recovery from exhaustive exercise appeared to be critically impaired above 16°C. The broad thermal range (~4–16°C) for increasing *f*_Hmax_ and maintaining absolute aerobic scope matches river temperatures commonly encountered by migrating Arctic char in this region. Nevertheless, river temperatures can exceed 20°C during warm events and our results confirm that such temperatures would limit exercise performance and thus impair migration in this species. Thus, unless Arctic char can rapidly acclimatize or alter its migration timing or location, which are both open questions, these impairments would likely impact population persistence and reduce lifetime fitness. As such, future conservation efforts should work towards quantifying and accounting for the impacts of warming, variable river temperatures on migration and reproductive success.

## Introduction

The Canadian Arctic has warmed at close to three times the average global rate, raising concern among northerners, scientists, and policymakers alike (Galappaththi *et al*., 2019; Zhang *et al*., 2019). Despite this concern, we know little about the environmental physiology of many Arctic species, even though such knowledge is fundamental to a mechanistic understanding of the ecological impacts of climate change (Cooke and O’Connor, 2010; Cooke *et al*., 2012; Cooke *et al*., 2013; Patterson *et al*., 2016). Indeed, knowledge regarding thermal limitations to metabolism, growth, and exercise performance is increasingly being used to develop evidence-based management strategies for many fishes (Cooke *et al*., 2012; DFO, 2012; Patterson *et al*., 2016). For instance, the allowable harvest of Pacific and Atlantic salmon is now adjusted based on river temperatures, in part, using knowledge that high temperatures, by impairing salmon cardiac performance, capacity for aerobic exercise, and recoverability, can reduce survival during migration (Farrell *et al*., 2008; Eliason *et al*., 2011; DFO, 2012; Patterson *et al*., 2016). Equivalent knowledge for use in management decisions remains sparse for Arctic fishes, largely due to the exorbitant cost of northern research (Mallory *et al*., 2018), technical limitations of conducting sensitive physiological research in remote field settings (Farrell *et al*., 2003; Mochnacz *et al*., 2017; Gilbert and Tierney, 2018), and the absence of field-based infrastructure available to support such research.

This limited physiological knowledge base is particularly concerning for keystone species such as the Arctic cod (*Boreogadus saida*; Drost *et al*., 2016) and for our focal species, the Arctic char (*Salvelinus alpinus*), which has immense cultural, subsistence and economic value to northern communities (Roux *et al*., 2011; Day and Harris, 2013; Roux *et al*., 2019). What little is known regarding wild Arctic char thermal physiology indicates that, like other anadromous salmonids, their thermal physiology likely varies with life stage, thermal history and population of origin. For instance, juvenile Arctic char in the central Canadian Arctic can maintain their exercise capacity over temperatures from 10 to 21°C but nevertheless struggle to recover from exhaustive exercise above 20°C (Gilbert and Tierney, 2018). In contrast, the maximum heart rate (*f*_Hmax_) of larger sea-run Arctic char in Greenland (presumably acclimated to sea surface temperatures of ~ 7°C) became thermally limited at just 13°C and the heart failed altogether by 15°C (Hansen *et al*., 2016); the optimal temperature for cardiorespiratory performance was estimated as ~ 7°C, based on the rate of increase in *f*_Hmax_ decreasing above 7°C. This temperature is well below the maximums seen in Canadian Arctic rivers through which Arctic char migrate in summer (Gilbert *et al*. 2016).

Anadromous Arctic char certainly experience dramatic shifts in available and experienced temperatures throughout their life history and broad, circumpolar distribution (Gilbert *et al*. 2016; Mulder *et al*., 2019; Harris *et al*. 2020). Juveniles typically rear in cool freshwater lakes (typically < 4°C; Godiksen *et al*., 2012) until they are large enough (~3–6 years old, > 180 mm; Johnson, 1989; Gilbert *et al*., 2016) to undertake a spring migration to their marine feeding environment where they spend most of their time at milder temperatures (~4–11°C; Rikardsen *et al*., 2007; Spares *et al*., 2012; Mulder *et al*., 2019; Harris *et al*., 2020), with occasional brief dives to frigid waters (−2 to 0°C). Unlike most anadromous salmonids, to avoid freezing over winter in seawater Arctic char must return to freshwater in late summer or fall and typically repeat this migration cycle many times throughout their life (Johnson, 1989; Gyselman, 1994; Klemetsen *et al*., 2003; Moore *et al*., 2016). Their up-river return migrations are among the greatest physical and thermal challenges in their lives (Gilbert *et al*., 2016). They can encounter conditions ranging from high-flow rapids to water so shallow that they are only partially submerged, and temperatures that can vary dramatically from ~ 0 to 21°C (present study; Gilbert *et al*., 2016; Gilbert and Tierney, 2018).

This variable thermal history, the rapid warming of the Arctic and inter-study differences in thermal tolerance provide the impetus for field-based research on the thermal physiology of Arctic char. To this end, we used innovative mobile Arctic research infrastructure and simplified physiological techniques to allow for a field-based assessment of the thermal limits to cardiorespiratory performance for migrating Arctic char in the central Canadian Arctic. Based on their range of encountered migration temperatures, we predicted that Arctic char in this region should have a relatively broad cardiorespiratory thermal performance and would tolerate temperatures warmer than those found in the only other comparable study of wild Arctic char (Hansen *et al*., 2016). The overall goal of the study is to provide insight into how current and future thermal regimes may impact Arctic char cardiorespiratory physiology and thus migration success in the face of rapid warming.

**Figure 1:**
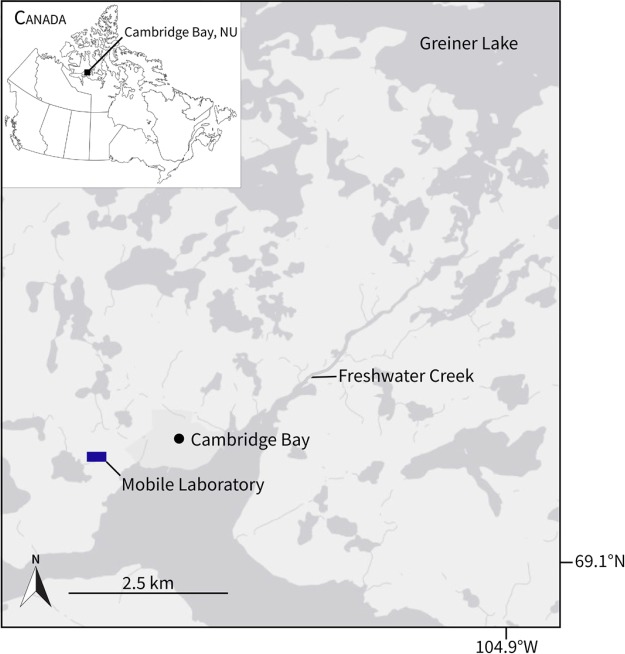
Study area around the hamlet of Cambridge Bay in the Kitikmeot region of Nunavut, Canada. Anadromous Arctic char were captured at the mouth or in the lower 850 m of Freshwater Creek, which drains the Greiner watershed into the Arctic Ocean.

## Methods

### Study animals and mobile research laboratory

All sampling and animal use was approved by Fisheries and Oceans Canada Freshwater Institute (AUP 2016-033, AUP 2017-039, LFSP S-16/17 1028-NU, LFSP S-17/18 1023-NU). In the summers of 2016 and 2017, anadromous Arctic char (2016: *n* = 20 length = 385 ± 13 mm (mean ± SEM), mass = 605 ± 61 g; 2017: *n* = 12 length = 407 ± 27 mm, mass = 711 ± 209 mm) were caught by angling with barbless hooks at the mouth or in the lower reach (850 m) of Freshwater Creek, NU, Canada (69°07′N, 104°59′W; Fig. 1), where there is an important subsistence fishery for Inuit from the community of Cambridge Bay. Captured fish were transported in an aerated 90-L cooler by all-terrain vehicle (ATV) to a nearby (<7 km) mobile research laboratory (Arctic Research Foundation, Winnipeg, CAN; Fig. S1) equipped with a 450-L temperature-controlled fish holding system (Aqua Logic Inc., San Diego, USA), where they were held at commonly encountered river temperatures (~10–12°C). The mobile laboratory was constructed out of a standard 6.1 × 2.4 × 2.4 m (20 × 8 × 8 feet) shipping container and equipped with 15 × 305 W solar panels, 2 × 1.1 kW wind turbines, a backup 10 kW diesel generator, 24 × 2 V batteries and 2 × 6.8 kW inverter and charging systems (Fig. S1). The laboratory was also fitted with bench space (~2 m^2^), equipment storage and a composting toilet.

### Respirometry and critical thermal maxima

In 2017, respirometry was conducted at temperatures from 3.7 to 20.0°C (*n* = 12). Arctic char were allowed to recover overnight (>12 h) following capture prior to these experiments. To begin, fish were warmed at 2°C h^−1^ to the test temperature and held at that temperature for 1 h. They were then chased to fatigue (time to fatigue: 4.4 ± 0.2 min), given 1.3 ± 0.1 min air exposure, during which a ~ 200 μL caudal blood sample was drawn, and the fish was rapidly sealed in a 30-L respirometer (90 cm long × 20.3 cm diameter). Fatigue was defined as the fish being refractory to a tail and mid-body grab for > 5 s. Fish were then allowed to recover for a minimum of 18 h in the respirometer after which they were warmed at rate of 5°C h^−1^ until they reached their critical thermal maximum (CT_Max_), the temperature at which they could no longer maintain dorsoventral equilibrium. This protocol allowed us to reliably estimate maximum (ṀO_2Max_) and minimum (ṀO_2Min_) oxygen consumption rates_,_ calculate absolute and factorial aerobic scope (AAS: ṀO_2Max_ − ṀO_2Min_; FAS: ṀO_2Max_/ṀO_2Min_), record MO_2_ during acute warming and estimate CT_Max_. AAS serves as a measure of the net aerobic capacity available above rest while FAS is a measure of the capacity to increase ṀO_2_ above ṀO_2Min_ in a multiplicative manner. Whether an aerobic function is constrained by AAS or FAS as temperature increases will depend on how its aerobic cost scales with temperature relative to the available scope (see Farrell, 2016; Hasley *et al*., 2018). Hematocrit (Hct), haemoglobin (Hb) content (HB 201, HemoCue, Ängelholm, SWE), blood glucose (Accu-Chek Aviva, Roche, Basel, CHE) and blood lactate concentration (Lactate Pro, Arkray KDK, JPN) were measured in duplicate immediately following the chase and at CT_Max_. Hb values were adjusted as previously described for fish (Clark *et al*., 2008) and mean corpuscular haemoglobin concentration (MCHC) was also calculated (Hb/Hct). Once the fish was sealed in the respirometer, intermittent flow respirometry was performed as previously described (Svendsen *et al*., 2016; Zhang *et al*., 2018) and the measurement and flush period varied based on the temperature, size of the fish and time post-chase to ensure an adequate ṀO_2_ signal was obtained (*R*^2^ > 0.8) and dissolved oxygen remained above 80% air saturation (Chabot *et al*., 2016). A full water change was conducted between each experiment to limit microbial load and waste accumulation. ṀO_2Max_ was estimated using an iterative algorithm to identify the steepest slope in dissolved oxygen over any measurement period (Zhang *et al*., 2018). ṀO_2Min_ was taken as the lowest 25% of the data recorded after 12 h of recovery (Chabot *et al*., 2016; Zhang *et al*., 2018). ṀO_2_ was recorded once every 12 min during warming from the test temperature at ~ 1°C increments as previously described (Gilbert *et al*., 2019).

### Heart rate assessment

Cardiac thermal tolerance was assessed in 2016 on anaesthetized fish fitted with electrocardiogram (ECG) electrodes in a manner similar to previous studies on other salmonids (Casselman *et al*., 2012; Anttila *et al*., 2014; Chen *et al*., 2015) and notably, in Greenlandic Arctic char (Hansen *et al*., 2016) and Arctic cod (Drost *et al*., 2016). To begin each experiment, two fish were anesthetized in 150 mg L^−1^ buffered tricaine methanesulfonate (TMS; 1:1.5 sodium bicarbonate) at 10°C until ventilation slowed, almost to a stop and was only intermittent. Fish were then weighed and transferred to a well-aerated anaesthetic bath containing 65 mg L^−1^ buffered TMS where they were placed supine, their gills were irrigated with a pump. Stainless steel electrodes were inserted into the skin over the heart on the right side of the ventral midline and just posterior to the left pectoral fin, and an ECG was acquired and processed as previously described (Gilbert *et al*., 2019). The fish were then given an intraperitoneal injection of 1.2 mg/kg atropine sulphate and 4 μg kg^−1^ isoproterenol in a total volume of 1 mL kg^−1^ 0.8% NaCl solution to stimulate their *f*_Hmax_. Once the heart rate stabilized (~20 min), the anaesthetic bath was warmed (*n* = 12) or cooled (*n* = 8) from 5 and 10°C, respectively, at a rate of 5°C h^−1^ in 1°C increments every 12 min using a drop-in coil heater–chiller (Isotemp II, Fisher Scientific, Hampton, USA), and a flow-through aquarium chiller (Tr15, Teco, Ravenna, ITA). The experiment continued until the heartbeat became arrhythmic or the bath began to freeze at which point the fish was euthanized with an overdose of anaesthetic (150 mg/L buffered TMS) followed by spinal pithing. The ECG was analyzed using automated heartbeat detection as in Gilbert *et al*. (2019). The temperature at the first Arrhenius break point (*T*_warmAB_), peak *f*_Hmax_, temperature at peak *f*_Hmax_ (*T*_peak_) and temperature at arrhythmia (*T*_arr_) were identified for each individual as previously described (Casselman *et al*., 2012; Chen *et al*., 2015; Hansen *et al*., 2016). We also identified an Arrhenius break point temperature (*T*_coldAB_) for each fish that was acutely cooled from 10°C. Briefly, Arrhenius break point temperatures were identified using a segmented linear regression and indicate a temperature at which there is a notable transition in the thermal sensitivity of a given rate with further warming or cooling. *T*_peak_ and peak *f*_Hmax_ are the temperature and *f*_H_ at which *f*_Hmax_ ceased to increase or began decreasing with further warming and thus indicate the *f*_Hmax_ and temperature at which a cardiac performance limitation occurred. *T*_arr_ is the temperature at which the heart began skipping beats (became irregular), thus indicating severe cardiac dysfunction.

### Analysis

All statistical analyses were conducted in R Studio (R Core Team, 2014) except for the segmented regression analysis which, along with all data presentation, was done using Prism v.8.3 (GraphPad Software, San Diego, USA). To account for allometric scaling, ṀO_2Max_, ṀO_2Min_ and ṀO_2_ during acute warming were adjusted to a common body mass by summing the residual values from the respective log(ṀO_2_) vs. log(mass) linear relationship with the predicated value at the average mass (0.71 kg). This adjustment was done prior to all other analyses. Based on relationships identified in previous studies (e.g. Eliason *et al*., 2013; Chen *et al*., 2015), the effect of test temperature on ṀO_2Max_ and ṀO_2Min_ was examined using linear and second-order polynomial models and the model with the lowest AICc value was presented. The effect of temperature (fixed effect) on ṀO_2_ and *f*_Hmax_ during acute warming was assessed using linear mixed effects models (LMMs; lme4 package; Bates *et al*, 2015) with Fish ID included as a random factor to account for the fact that multiple measurements were made on each individual. The *f*_Hmax_ model was restricted to values recorded below an individual’s *T*_peak_. Test statistics for LMM were generated using Satterthwaite’s degrees of freedom method in the ‘lmertest’ package (Kuznetsova *et al*., 2017) and marginal and condition correlation coefficients were calculated using the ‘MuMIn’ package (Barton and Barton, 2015). The normality of model residuals was confirmed using the Shapiro–Wilk test. Arrhenius break point temperatures were determined during cooling and warming as previously described (Casselman *et al*., 2012; Chen *et al*., 2015). All data are presented as mean ± SEM unless otherwise noted and *α* = 0.05.

**Figure 2:**
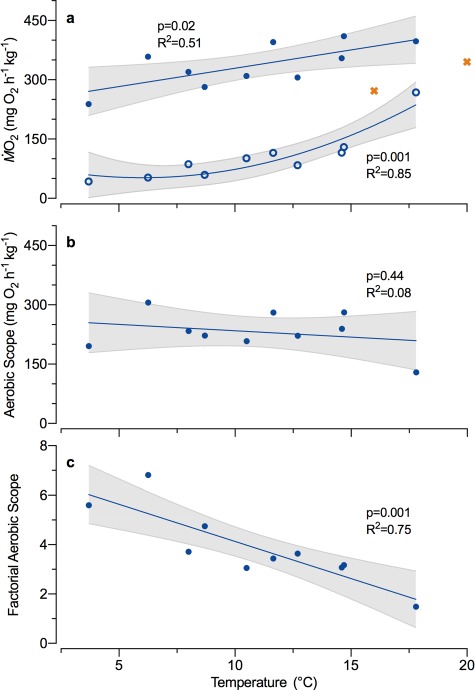
The effect of environmentally relevant temperatures on (**a**) minimum (ṀO_2Min_; open circles; *n* = 10) and maximum oxygen uptake (ṀO_2Max_; closed circles; *n* = 12) and aerobic scope of migratory Arctic char following a chase to exhaustion and air exposure. (**b**) Absolute aerobic scope (AAS) and (**c**) factorial aerobic scope (FAS). Two individuals at warm temperatures died after being exhausted (orange ‘x’) and were excluded from all regression analyses. A linear model and a second-order polynomial model were compared for each variable. The model with the lowest AICc is presented (blue lines) with its 95% confidence interval (shaded area), *R*^2^ and *P* value.

## Results

### Respirometry and CT_Max_

River temperatures ranged from 8.9 to 14.5°C (mean ± SD: 11.0 ± 1.4°C; recorded every 15 min) during the period when fish were captured for the respirometry experiments. Arctic char were acutely brought (2°C h^−1^) to their test temperature (*T*) before being chased to fatigue, which lasted 4.4 ± 0.2 min, independent of temperature (*f*_1,10_ = 0.49, *P* = 0.50, *R*^2^ = 0.05). ṀO_2Max_ increased linearly with increasing test temperature (Fig. 2a; Eq. 1, *f*_1,8_ = 8.3, *P* = 0.02, *R*^2^ = 0.51), while ṀO_2Min_ increased exponentially (Fig. 2a; Eq. 2, *f*_2,7_ = 19.9, *P* = 0.001, *R*^2^ = 0.85). Even so, AAS did not change significantly with increased temperature (Fig. 2b; Eq. 3, *f*_1,8_ = 0.65, *P* = 0.44, *R*^2^ = 0.08), whereas FAS decreased markedly (Fig. 2c; Eq. 4, *f*_1,8_ = 23.6, *P* = 0.001, *R*^2^ = 0.75).

(1) \begin{equation*} \dot{\mathrm{M}}{\mathrm{O}}_{2\operatorname{Max}}=9.6\pm 3.0\bullet T+231.9\pm 35.0 \end{equation*}

(2) \begin{equation*} \dot{\mathrm{M}}{\mathrm{O}}_{2\operatorname{Min}}=1.2\pm 0.4\bullet{T}^2-12.4\pm 8.1\bullet T+80.5\pm 40.9 \end{equation*}

(3) \begin{equation*} \mathrm{AAS}=-3.0\pm 3.9\bullet T+265.9\pm 45.6 \end{equation*}

(4) \begin{equation*} \mathrm{FAS}=-0.34\pm 0.06\bullet T+7.71\pm 0.65 \end{equation*}

Importantly, two of the three fish exercised to exhaustion above 15°C died during recovery and had a lower ṀO_2Max_ than would be predicted based on the linear model for the remaining fish (Fig. 2a). The individual that did recover after being chased at > 15°C exhibited the lowest AAS and CT_Max_ and had the highest blood lactate concentration (15.1 mM) post-CT_Max_. Whether this elevated lactate was a result of the notably elevated ṀO_2Min_ (Figs 1a and 2) or a more severe degree of exhaustion is unclear.

**Figure 3:**
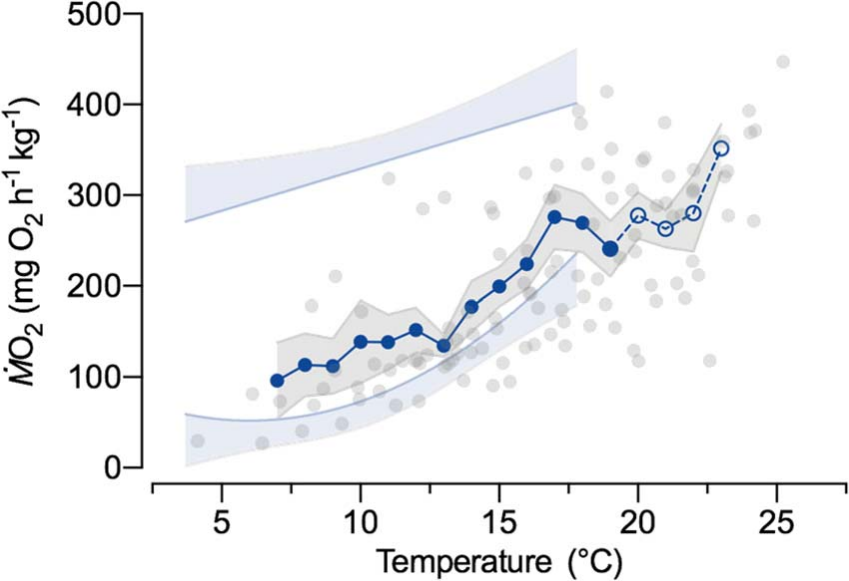
Oxygen uptake (ṀO_2_) of anadromous Arctic char during acute warming to loss of equilibrium (CT_Max_). Individual ṀO_2_ values at their measured temperatures (grey circles; *n* = 8 individuals and 111 data points) and mean ± sem values in 1°C bins (blue circles and grey shading) are presented. Mean values were calculated at all points where data were available for three or more individuals. The dashed line indicates temperatures where individuals were removed after reaching CT_Max_. For reference, the models for maximum ṀO_2_ and ṀO_2Min_ (Fig. 1) are presented with their upper or lower 95% confidence intervals, respectively (light blue lines with shaded areas).

Acute warming following the post-chase recovery period doubled ṀO_2_ (LMM: *f*_1,8.9_ = 145.1, *P* < 0.001, marginal *R*^2^ = 0.50, conditional *R*^2^ = 0.83) from 138.8 ± 37.2 mg O_2_ h kg^−1^ at 10°C to 277.7 ± 22.0 mg O_2_ h kg^−1^ at 20°C (*Q*_10_ = 2.0). Peak ṀO_2_ during acute warming was 319.1 ± 33.6 mg O_2_ h kg^−1^ (at 19.8 ± 1.1°C), a value similar to that measured immediately after exhaustive exercise at warm temperature (Figs 2a and 3). Likewise, a similar ṀO_2_ (291.1 ± 42.7 mg O_2_ h kg^−1^) also was seen immediately prior to reaching CT_Max_ (23.0 ± 0.6°C; range 19.0 to 25.2; Fig. 5).

**Figure 4:**
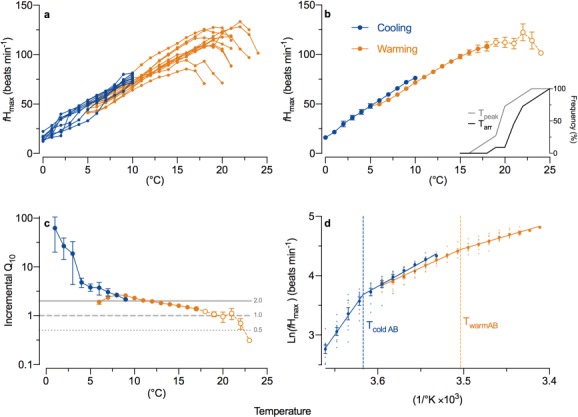
Maximum heart rate (*f*_Hmax_) of anadromous Arctic char during acute warming from 5°C (orange; *n* = 12) and cooling from 10°C (blue; *n* = 8) as (**a**) individual and (**b**) average responses. The cumulative proportion of individuals that reached their temperature at peak *f*_Hmax_ (*T*_peak_) and arrhythmia (*T*_arr_) are inset (**b**). The thermal sensitivity of *f*_Hmax_ is shown using (**c**) the temperature coefficient (*Q*_10_) calculated over 2°C increments, with reference lines indicating rates of change that would correspond to a doubling (2.0), plateau (1.0) or halving (0.5) of *f*_Hmax_ over 10°C and (**d**) an Arrhenius plot of *f*_Hmax_ showing the first Arrhenius break points during warming (*T*_warmAB_) and cooling (*T*_coldAB_). Averaged data (b–d) are presented ± sem and dashed connecting lines with open circles indicate temperatures where some individuals were removed from the analysis following arrhythmia.

### *f*_Hmax_ and cardiac thermal tolerance

**Figure 5:**
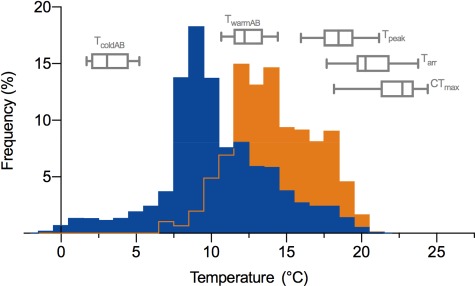
Transition temperatures for maximum heart rate (*f*_Hmax_) and the critical thermal maximum (CT_Max_) for anadromous Arctic char relative to frequency distributions for water temperatures during their upriver migration. Arrhenius break point temperatures (cooling: *T*_coldAB_ warming: *T*_warmAB_) and temperatures at peak *f*_Hmax_ (*T*_peak_), onset of arrhythmia (*T*_arr_) and loss of equilibrium (CT_Max_) are shown relative to the average (blue) and warmest (orange; Gilbert *et al*. 2016) river temperature frequencies recorded during upriver migrations in the Kitikmeot region of Nunavut, Canada. River temperatures were compiled for four rivers from past (Gilbert *et al*. 2016) and ongoing fisheries research (M.J.H.G. and L.N.H. unpublished data). Boxes represent the median and interquartile range and whiskers indicate the 5th and 95th percentile of data.

River temperatures ranged from 9.7 to 12.4°C (mean: 10.6 ± 0.24) at the site of capture of fish used for the cardiac thermal tolerance experiments. *f*_Hmax_ in Arctic char acutely transferred to 5°C was 47.7 ± 1.3 beats min^−1^. Acute warming of these fish increased their *f*_Hmax_ (LMM: *f*_1,9.6_ = 807.5, *P* < 0.001, marginal *R*^2^ = 0.92, conditional *R*^2^ = 0.98), attaining a peak *f*_Hmax_ of 115.4 ± 4.7 beats min^−1^ at 19.4 ± 0.5°C (*T*_peak_; Figs 4 and 5). Above T_peak_, *f*_Hmax_ declined to 99.0 ± 5.9 beats min^−1^ and became arrhythmic at 21.4 ± 0.5°C (*T*_arr_; Figs 4 and 5) before the experiment was terminated. The temperature of the first Arrhenius break point in *f*_Hmax_ during warming (*T*_warmAB_) was 12.5 ± 0.3°C (Fig. 4a and b), which was similar to common ambient river temperatures (Fig. 5). The instantaneous *Q*_10_ for *f*_Hmax_ decreased progressively with warming (Fig. 4c), falling below 2.0 at ~ 11°C; the *Q*_10_ of 1.0 at ~ 19°C indicated that, on average, *f*_Hmax_ had peaked or plateaued (Fig. 4c). With acute cooling from 10 to 0°C, *f*_Hmax_ progressively decreased from 76.3 ± 1.3 beats min^−1^ to 16.1 ± 1.2 beats min^−1^ (LMM: *f*_1,7.0_ = 1273.9, *P* < 0.001, marginal *R*^2^ = 0.93 conditional *R*^2^ = 0.97) and exhibited a clear break point at ~ 3.3 ± 0.5°C (Fig. 4d), below which the thermal dependence of *f*_Hmax_ increased sharply with an instantaneous *Q*_10_ > 10 (Fig. 4c).

### Blood properties

**Figure 6:**
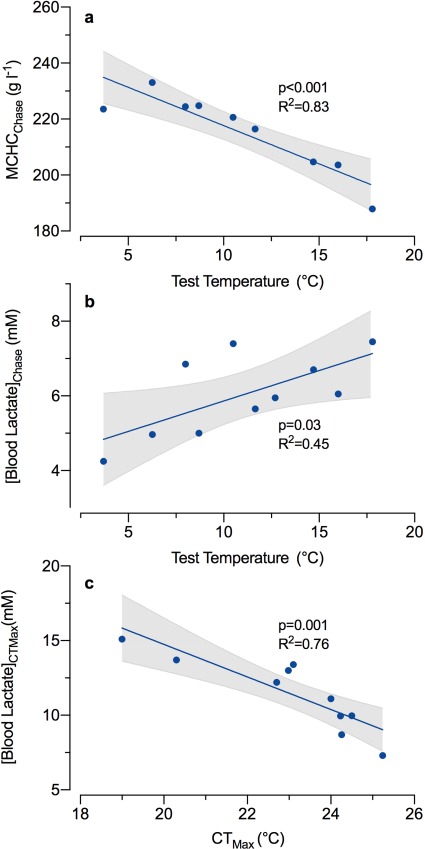
Correlations between blood properties and either the test temperature or the critical thermal maximum (CT_Max_). Blood was drawn immediately following the chase to exhaustion (**a** and **b**) or immediately after fish lost equilibrium at their CT_Max_ (**c**). Significant relationships were identified through a pairwise Spearmen’s correlation analysis (*α* = 0.05; Fig. S2). Relationships for mean corpuscular haemoglobin concentration (MCHC; a) and blood lactate (b and c) are shown with their corresponding linear model (solid line) and with 95% confidence intervals (dashed line).

The MCHC decreased (Fig. 6a; *f*_1,7_ = 33.3, *P* < 0.001, *R*^2^ = 0.83) and blood lactate concentration increased (Fig. 6b; *f*_1,8_ = 6.6, *P* = 0.03, *R*^2^ = 0.45) with test temperature when measured immediately after Arctic char were chased to exhaustion. Blood lactate measured immediately after CT_Max_ was negatively correlated with CT_Max_ (Fig. 6c; *f*_1,8_ = 21.2, *P* = 0.001, *R*^2^ = 0.76). Additional blood parameters were correlated with each other, but not with the test temperature or CT_Max_ (Fig. S2).

## Discussion

Anadromous Arctic char can encounter a broad range of temperatures from ~ 0 to 21°C during their physically demanding, upriver migrations in the Canadian Arctic (Fig. 5; Gilbert *et al*., 2016) and even lower when at sea (Harris *et al*., 2020). Here we show that wild migrating Arctic char can impressively maintain absolute aerobic scope and a high regular heart rate over a large proportion (~4–16°C) of this thermal range. However, factorial aerobic scope (FAS), their ability to recover from exhaustive exercise and their *f*_Hmax_ can become critically limited > 16°C, temperatures that are well within current extremes (Fig. 5). Moreover, and unexpectedly, *f*_Hmax_ was greatly depressed with acute cooling to 0°C. As we discuss below, these traits may be intimately linked to the need for Arctic char to repeatedly perform prolonged swimming bouts over a wide range of temperatures in order to complete their migrations and successfully reproduce. Importantly, our findings were made possible by the use of innovative mobile research infrastructure (Fig. S1). Thus, the present study demonstrates the critical importance of such infrastructure and logistical support in any effort to understand pressing conservation and management issues in remote Arctic locations.

### Cold performance

Early research on cold-adapted fish species suggested that they may exhibit elevated metabolic rates at cold temperatures to allow elevated growth and activity when compared to related species that were not cold-adapted (Scholander *et al*., 1953; Wohlschlag, 1960). This idea, which was termed ‘metabolic cold adaptation’, has largely been disproven and currently has limited support (Holeton, 1973; Holeton, 1974; Steffensen *et al*., 1994; Steffensen, 2002; White *et al*., 2012). Indeed, when Holeton (1973) tested this hypothesis on wild juvenile Arctic char in the Canadian Arctic, he found that their resting ṀO_2_ was no higher than would be predicted for more temperate salmonids despite the Arctic char being adapted and acclimatized to a cold environment. However, Holeton’s measurements of resting ṀO_2_ measurements at ambient temperatures (2°C) were technically limited by available equipment, facilities and technology. Although our ṀO_2_ data do not extend to 0°C, our ṀO_2_ data for cool temperatures similarly indicate that Arctic char do not exhibit elevated metabolism when compared with other salmonids (e.g. Hvas *et al*., 2017). Instead, *f*_Hmax_ clearly and sharply declined (high *Q*_10_) at < 3.3°C, suggesting the potential for Arctic char to undergo metabolic rate suppression rather elevation metabolism at frigid temperatures. Further, we propose this transition in *f*_Hmax_ may be part of a suite of behavioural and physiological overwintering strategies that conserve energy during a period of limited food availability. Indeed, other species display an active depression of metabolic rate (Guppy and Withers, 1999), fasting and suppression of activity (Speers-Roesch *et al*., 2018). However, as we did not directly characterize acute or prolonged responses of ṀO_2_ to cold temperature (<3°C), this possibility remains to be tested.

While the observed limitation in *f*_Hmax_ at cold temperatures could be part of an adaptive energy conservation strategy, it would likely limit maximum cardiac output and thus aerobic exercise capacity. This cold limitation could, in turn, impact performance during demanding activities such as river migrations or diving down to frigid waters to forage. Indeed, telemetry data suggest that dives to colder, deeper ocean waters are typically very brief (Spares *et al*., 2012; Harris *et al*., 2020). If this is indeed an issue, cold acclimation could help mitigate the observed effects of acute cold exposure. Fish in the present study were presumably acclimated to near the ambient river temperatures at which they were caught (~11°C), while Arctic char at sea (e.g. ~ 5–8°C, Harris *et al*., 2020) or overwintering (e.g. 0.5–2°C; Mulder *et al*., 2018) would be acclimated to colder water. Indeed, the only other comparable study of *f*_Hmax_, which focused on ocean-caught Arctic char, did not find such a pronounced change in *f*_Hmax_ at cold temperatures (Hansen *et al*., 2016). Furthermore, cold acclimation has previously been shown to increase intrinsic and maximum *f*_H_ in many fishes, which helps counteract rate limitations inherently associated with cold exposure (Aho and Vornanen, 2001; Drost *et al*., 2016; Eliason and Anttila, 2017).

### Warm performance

While debate continues over the ecological relevance of CT_Max_ and similar acute lethal thermal tolerance measures (Pörtner and Peck, 2010; Sunday *et al*., 2011), such metrics remain useful, relative indicators of whole-organism heat tolerance and are unarguably the ceiling for thermal performance (Sandblom *et al*., 2016). Previous measures of CT_Max_ in hatchery-reared (~0.02–0.05 kg) European Arctic char parr (e.g. ~ 26–28°C; Baroudy and Elliott, 1994; Anttila *et al*., 2015) are significantly higher than CT_Max_ for adults in the present study (23°C). In contrast, CT_max_ for adult Arctic char (0.7 kg) reared in a marine aquaculture in eastern Canada and acclimated to ~ 10°C (23°C; Penney *et al*., 2014) was identical to the present result. By comparison, adult Arctic cod, a classic polar stenotherm and important food source for Arctic char, have a much lower CT_Max_ (~15–17°C, Drost *et al*., 2016), whereas the CT_Max_ of adult rainbow trout (0.3–0.7 kg) and Atlantic salmon (0.7 kg), both temperate relatives of Arctic char, is notably higher (~26–27°C; Ekström *et al*., 2014; Penney *et al*., 2014; Gilbert *et al*., 2019). In the present study, elevated blood lactate was associated with a lower CT_Max_, indicating that anaerobic stress prior to or during acute warming may subsequently decrease heat tolerance, which could be particularly important for exercising (migrating)fish.

Sub-lethal thermal limitations to physiological performances, such as a collapse in aerobic scope and heart rate, or cardiac arrhythmia, are arguably of greater ecological relevance than acutely lethal limitations because they occur at lower, more commonly encountered temperatures (Fig. 4). Penney *et al*. (2014) monitored *f*_H_ and ṀO_2_ in Arctic char during acute warming and found an identical peak *f*_H_ to that found here (115 beats min^−1^). This similarity suggests that acutely warmed adult Arctic char reach their physiological maximum *f*H prior to CT_Max_, leaving little to no scope for *f*_H_ available to support further warming. At 10°C the same study (Penney *et al*., 2014) found that resting *f*_H_ was ~ 44 beats min^−1^ whereas *f*_Hmax_ at 10°C was ~ 74 beats min^−1^ in the present study, highlighting a significant scope to increase *f*_H_ at cooler temperatures. Despite the similarities, Penney *et al*. (2014) found that the peak ṀO_2_ achieved during warming was only 223 mg O_2_ h kg^−1^ compared to ~ 319 mg O_2_ h kg^−1^ in the present study, which may indicate a loss of aerobic performance following captive rearing or domestication, as found in Atlantic salmon (Zhang *et al*., 2016).

Hansen *et al*. (2016) examined cardiac heat tolerance in sea run char from Greenland and found that *T*_warmab_, *T*_peak_, *T*_arr_ and peak *f*_Hmax_ were only 7.5, 12.8, 15.2 and 61.8 beats min^−1^, respectively. All of these thermal performance indicators are all markedly lower (*T*_warmab_: −5.0°C *T*_peak_: −6.6°C, *T*_arr_: −6.2°C and peak *f*_Hmax_: −53.6 beats min^−1^) than in the present study, despite similar methodology and fish size. There were, however, key differences between the studies including the life history period (marine vs. upstream fall migrating), fish provenance (Greenland vs. central Canadian Arctic) and possibly acclimation temperatures (~7 vs ~ 11°C). The most parsimonious explanation for these differences is the potential for either thermal acclimation or thermal adaptation among populations (as discussed below).

Gilbert and Tierney (2018) found that acutely warmed wild smolts and lab-reared juvenile Arctic char (~0.1 kg) maintained swimming performance up to 21°C, but their recovery was impaired above > 20°C. This impaired recoverability was associated with highly elevated blood lactate concentrations, which can be indicative o increased anaerobic demand and metabolic acidosis. Here, blood lactate levels also increased with temperature and we observed impaired recoverability following exhaustive exercise albeit in much larger animals at an even lower temperature (>15–16°C). We measured blood lactate immediately following chasing although lactate release from tissues can continue over a longer time course (Milligan, 1996). As such, our results could be a product of increased rate of lactate release into the blood rather than elevated anaerobic metabolism. However, we also observed a decrease in MCHC with increased temperature, which is indicative of red blood cell swelling in response to metabolic acidosis (Nikinmaa *et al*., 1987). Gilbert and Tierney (2018) also found that a handling challenge in large migrating adult Arctic char (~4 kg) resulted in an increase in reflex impairment above 12°C, which was associated with early mortality. These post-handling impairments were particularly severe (>50%) above 15°C, which is lower than their observations in smolts (>20°C) and consistent with results for adults in the present study. Together these data suggest that access to cold-water refugia in lakes or pools may be critical for recovery if they encounter and can swiftly pass through warm temperatures during their up-river migration.

Arctic char are clearly more heat tolerant than classically stenothermal polar fishes, likely as a result of the extreme thermal variability they can encounter over the course of their lives (Janzen, 1967; Verde *et al*., 2008). Nonetheless, they are among the least heat-tolerant salmonids (Elliott and Elliott, 2010; Penney *et al*., 2014; Gilbert and Tierney, 2018), yet they are already encountering temperatures warm enough to impair vital physiological functions and impact survival. Given that fish only recruit maximum physiological performances (e.g. MO_2Max_ and *f*_Hmax_) during demanding activities (e.g. navigating rapids) and operate at a sub-maximal performance level most of the time, further research is needed to examine temperature effects on sub-maximal performances such as routine swimming, growth, feeding and digestion.

### Future directions: examining sources of variation in Arctic char thermal physiology

The inter-study differences in thermal physiology highlighted above suggest that thermal history, ontogeny, and genetic background likely all contribute to thermal performance in Arctic char in a similar manner to other salmonids. For example, in Atlantic salmon warm acclimation can markedly increase cardiac thermal tolerance (Anttila *et al*., 2014). However, comparable knowledge for Arctic char is almost completely lacking. Ontogeny also has a pronounced effect on thermal physiology (Elliott and Elliott, 2010; Portner *et al*., 2017; Hines *et al*., 2019). Such effects have not been well characterized in Arctic char but likely contribute to incongruences between the present and past studies (see Larsson, 2005; Larsson and Berglund, 2005; Mortensen *et al*., 2007; Mulder *et al*., 2019; Harris *et al*. 2020).

In temperate salmonids (e.g. *Oncorhynchus* spp.), thermal tolerance tends to be higher in strains from warm habitats (Rodnick *et al*., 2004; Chen *et al*., 2015; Verhille *et al*., 2016; Poletto *et al*., 2017). Based on the current literature, European Arctic char may appear more thermally tolerant than Arctic char from Greenland and the Canadian Arctic. European Arctic char are from a different glacial lineage than Greenlandic and northern Canadian Arctic char and have therefore evolved independently for > 250 000 years (Brunner *et al*., 2001; Moore *et al*., 2015). This isolation may provide a genetic basis for regional differences in thermal physiology. Even within a watershed, the cardiorespiratory thermal performance curves of sockeye salmon (*O. nerka*) appear locally adapted to the conditions they encounter during their upriver migration (Eliason *et al*., 2011). While a recent study has shown that Kitikmeot Arctic char exhibit genetic differences among populations that are consistent with the hypothesis that they may be locally adapted (Moore *et al*., 2017), no study to date has examined this at the phenotypic level.

### Conclusions: thermal barriers to migration

Extreme heat events have repeatedly been shown to impose thermal barriers on the migration of temperate salmonids (Baisez *et al*., 2011; Martins *et al*., 2011; Hinch *et al*., 2012; Martins *et al*., 2012) by critically impairing physiological performances (Cooke *et al*., 2006; Farrell *et al*., 2008; Farrell, 2009; Eliason *et al*., 2011). Here we clearly show that, like these temperate salmonids, migrating Arctic char experience limitations in cardiac performance, FAS and recoverability at water temperatures that already occur during their migrations in some Arctic rivers (Fig. 4; Gilbert *et al*., 2016). Such limitations can critically impair migration, and they are only going to become more common as the Canadian Arctic is among the most rapidly warming regions on our planet (Zhang *et al*., 2019). The consequences of migration failure and associated reductions in fitness may be particularly dire in regions like the Kitikmeot, NU, where Arctic char are heavily harvested and relied upon as both a subsistence and economic resource (Day and Harris, 2013; Roux *et al*., 2019). As such, understanding and mitigating the impacts of extreme temperature events should be an urgent priority for fisheries managers, researchers and conservationists alike.

## Funding

This research was funded by Polar Knowledge Canada through the Science and Technology Program (2017) and the Northern Scientific Training Program (M.J.H.G.) as well as by the Natural Sciences and Engineering Research Council through Discovery and Canada Research Chair awards (A.P.F.) and an Alexander Graham Bell Canada Graduate Scholarship (M.J.H.G.).

## Supplementary Material

TTAC_MS_Revised_coaa036
