# Supplementary material for: The thermal limits of cardiorespiratory performance in anadromous Arctic char (Salvelinus alpinus): a field-based investigation using a remote mobile laboratory
Source: Conserv Physiol. 2020 Apr 23;8(1):coaa036. doi: 10.1093/conphys/coaa036 (PMC7176916; doi:10.1093/conphys/coaa036)

**Figure S1.** The Arctic Research Foundation mobile laboratories staged near Cambridge Bay, Nunavut, Canada. Photos depict (a) the solar arrays and turbines on three laboratories, (b) the turbines, entrance and generator for the marine and aquatic laboratory used in the present study, and (c) cardiac thermal tolerance experiments being conducted inside the laboratory. The shipping containers used to construct the laboratories are 6.1×2.4×2.4m (20×8×8’).


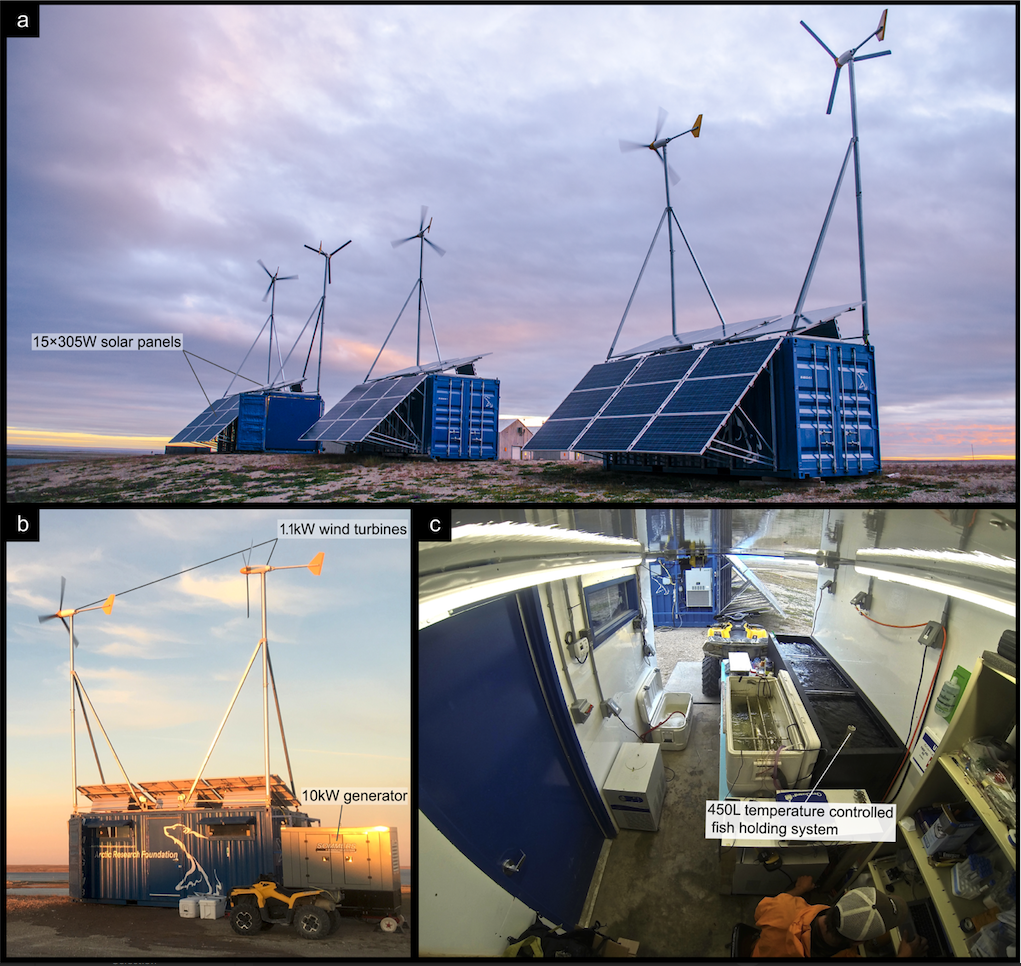


**Figure S2.** Correlation matrix for test temperature, critical thermal maximum (CT_Max_) and all measured blood parameters. Blood parameters include Glucose, Lactate, haemoglobin (Hb), hematocrit (Hct) and mean corpuscular haemoglobin content (MCHC) immediately following chasing or CT_Max_. Blood was sampled immediately following the chase to exhaustion (chase) or immediately after fish lost equilibrium at their CT_Max_ (CT_Max_). Spearman’s correlation coefficients are presented above the dashed line and p-values are below.


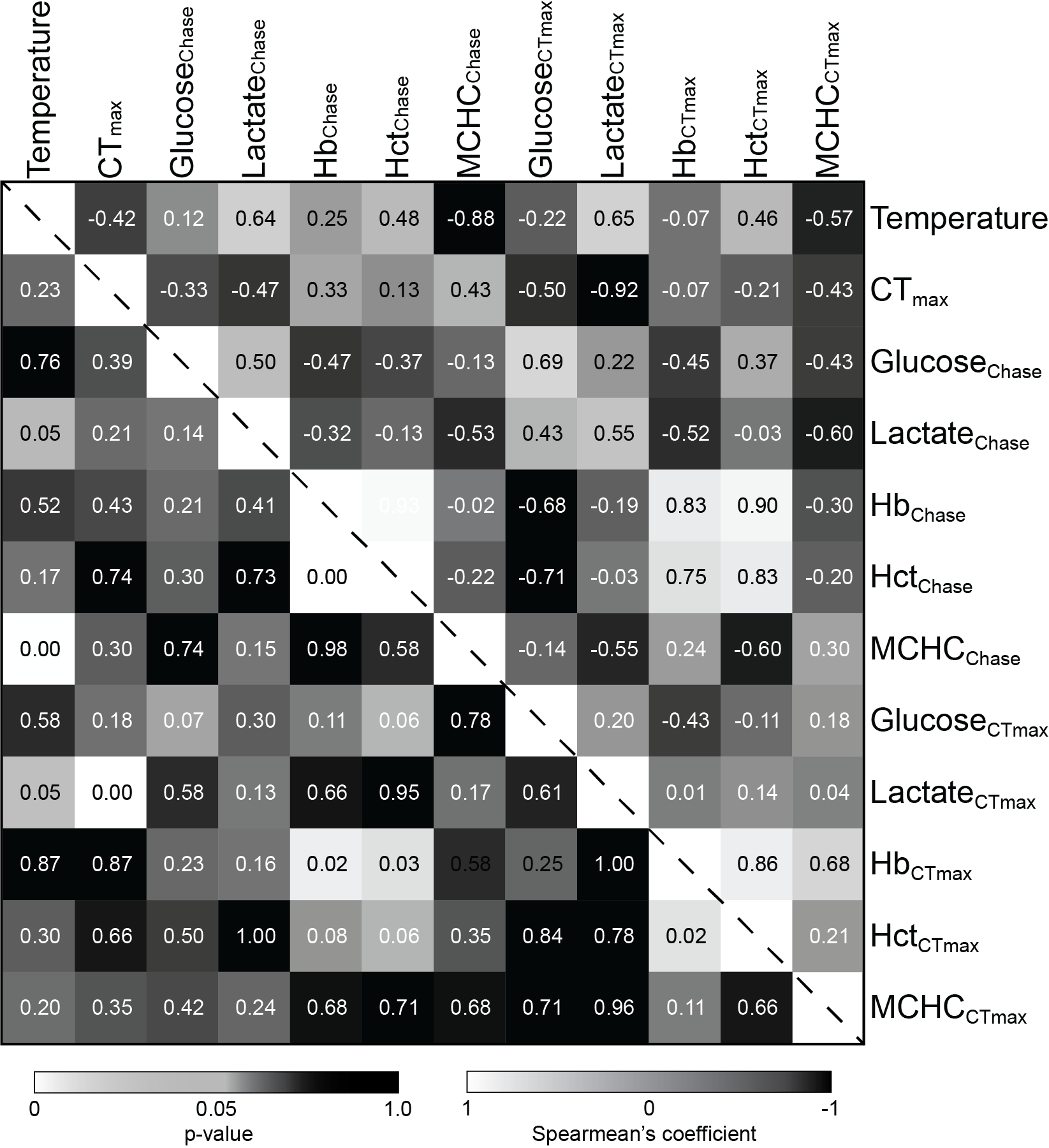

Supplement: TTAC_MS_Revised_coaa036 [file ttac_ms_revised_coaa036.docx]
